# Health benefits of docosahexaenoic acid and its bioavailability: A review

**DOI:** 10.1002/fsn3.2299

**Published:** 2021-07-23

**Authors:** Jia Li, Bernard L. R. Pora, Ke Dong, Jovin Hasjim

**Affiliations:** ^1^ Roquette Management (Shanghai) Co., Ltd. R&D China Shanghai China

**Keywords:** bioavailability, cardiovascular disease, cognitive function, docosahexaenoic acid, polyunsaturated fatty acids, prebiotics

## Abstract

Docosahexaenoic acid (DHA) is the predominant omega‐3 long‐chain polyunsaturated fatty acid found in human brain and eyes. There are a number of studies in the literature showing the health benefits of DHA. It is critical throughout all life stages from the need for fetal development, the prevention of preterm birth, and the prevention of cardiovascular disease to the improvements in the cognitive function and the eye health of adults and elderly. These benefits might be related to the modulation of gut microbiota by DHA. In addition, there are some discrepancies in the literature regarding certain health benefits of DHA, and this review is intended to explore and understand these discrepancies. Besides the variations in the DHA contents of different supplement sources, bioavailability is crucial for the efficacy of DHA supplements, which depends on several factors. For example, DHA in phospholipid and triglyceride forms are more readily to be absorbed by the body than that in ethyl ester form. In addition, dietary lipids in meals and emulsification of DHA oil can increase the bioavailability of DHA. Estrogens stimulated the biosynthesis of DHA, whereas testosterone stimulus induced a decrease in DHA. The roles of DHA through human lifespan, the sources, and its recommended daily intake in different countries are also discussed to provide a better understanding of the importance of this review.

## Graphical abstract

DHA is critical for all life stages from fetal development to elderly health. The recommended intake of DHA depends on many factors, such as regions, age groups, and genders. DHA supplements are mainly from fish, krill, and algal oils, and the bioavailability of DHA depends on its chemical structure and matrix.

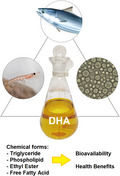

## 1 INTRODUCTION

Docosahexaenoic acid (22:6*n*‐3, DHA) and eicosapentaenoic acid (20:5*n*‐3, EPA) belong to the omega‐3 (*n*‐3) long‐chain (LC) polyunsaturated fatty acids (PUFA). Out of the two, DHA is the most important and the most abundant *n*‐3 PUFA in the brain, whereas only a small amount of EPA has been detected. High contents of DHA have been identified in the gray matter of brain (up to 20% of its total lipids) and in the outer rod segments of retina. DHA contributes to approximately 97% and 93% of the *n*‐3 PUFA in these organs, respectively (Lauritzen et al., 2001; Tambakhe & Pawar, 2014). Furthermore, it is one of the key and essential components of neural tissues (Kidd, 2007) and membrane structure (such as the synaptic terminals; Swanson et al., 2012). DHA also functions as the precursors of several metabolites (Ghasemifard et al., 2014; Serhan et al., 2008) and the modulators of central and enteric nervous system (Morena et al., 2015). In addition, DHA involves in the anti‐inflammatory and physiological processes, and it affects the cellular characteristics, such as membrane fluidity and neuronal differentiation and growth.

A large number of studies have demonstrated that dietary DHA has numerous health benefits throughout human life, including brain and eye developments of fetuses and infants (Drover et al., 2011; Dunstan et al., 2008; Judge et al., 2007; Willatts et al., 2016), prevention of early preterm delivery (Harris et al., 2015; Kar et al., 2016; Knudsen et al., 2006; Olsen et al., 2000), prevention of cardiovascular disease (CVD; Allaire et al., 2016; Asztalos et al., 2016; Bernstein et al., 2012; Mori, Burke, et al., 2000), and improvements in the cognitive (Stonehouse et al., 2013; Yurko‐Mauro et al., 2015) and the eye health (Christen et al., 2011; Chua et al., 2006; Seddon et al., 2006) of adults and elderly. New research studies have also shown that the benefits of dietary DHA might be related to the modulation of gut microbiota (Fu et al., 2021; Younge et al., 2017).

Experts have set the daily recommendations for *n*‐3 PUFA intake, in particular DHA. According to the 2010 US dietary guidelines, it is recommended to consume eight ounces of a variety of seafood per week (equivalent to 250 mg DHA and EPA per day) in order to prevent CVD (Marshall, 2011). For pregnant and lactating women, the recommended consumption is about 200 mg/day of DHA alone in most countries (Sposito et al., 2007), which is higher than 250 mg of the combination of DHA and EPA per day for normal adults. Similarly, there are specific recommended daily intakes (RDI) of *n*‐3 PUFA, in particular DHA, for different age groups, but they are different from one country to another, which can be attributed to many factors, such as hormones, dietary habits, and environment.

Furthermore, it is a challenge for consumers to reach the RDI of DHA through diet alone. There is still a big gap between current dietary intake and the recommended intake set by the experts in the world. The results of a global survey (Marshall, 2011) indicated that only 24% of the world adult population met the *n*‐3 fat level set in the 2010 US guidelines from the consumptions of seafood alone, while 67% had intake of less than 100 mg *n*‐3 fats/day, especially in China, which was less than 50 mg *n*‐3 fats/day (Micha et al., 2014). Therefore, DHA supplement is crucial and its consumption has increased worldwide. The most common source of DHA supplementation is fish oil. Krill oil is another marine source of DHA. In recent decades, algal oil has become an important source of DHA supplementation due to its good sustainability and the absence of marine pollutants. These oils are normally present in triglyceride (TG) or phospholipid (PL) forms. The *n*‐3 PUFA supplements with higher DHA levels are also commercially available in ethyl ester (EE) form. However, the bioavailability of DHA is affected by their lipid structure (Davidson et al., 2012; El Boustani et al., 1987; Lawson & Hughes, 1988b). This is not commonly discussed, which may lead to the discrepancies observed in the studies related to the health benefits of DHA supplementation, such as those reported by Ulven et al. (2011), Tou et al. (2011), and Köhler et al. (2015). In addition, the embedding matrix of DHA, such as the food matrix and the excipient, can alter the bioavailability of DHA (i.e., the absorption, the excretion, and the retention in the tissues; Rauch et al., 2010).

This narrative review is intended to explore the discrepancies found in the literature regarding the health benefits of DHA supplementation and to understand the factors that alter the bioavailability of DHA. The roles of DHA throughout human lifespan, its sources, and its RDI are also discussed to provide a better understanding on the importance of this review. Many previous reviews were mostly focused on the functions of *n*‐3 LCPUFA in general, rather than the specific roles of DHA. The outcome of this review can serve as a good foundation for study design of future research and development of DHA, including its applications.

## 2 HEALTH BENEFITS OF DHA

### 2.1 Fetal and infant development

DHA is one of the key nutrients in the development and the maturity of brain and eyes for infants. There is a spurt of DHA deposition in the brain during the last trimester of gestation and during the first 2 years of infancy, along with the rapid brain growth. The deposition rate of DHA slows down after infancy. The DHA content reaches the maximum in early adulthood and then gradually decreases with the aging process (Carver et al., 2001).

Half of the brain DHA is accumulated during the gestation period, where a developing infant brain requires five times the lipid level per day of an adult brain (Haag, 2003). As the biosynthesis of DHA from its precursors in the fetus and the placenta is not sufficient to meet the demand of the rapidly developing neural tissues (Uauy et al., 2000), the maternal DHA, which is dependent on the dietary intake of the mother, becomes an important factor for the fetal brain development (Grantham‐McGregor et al., 2007). Multiple benefits of DHA for infants have been demonstrated, including improved hand‐eye coordination (Dunstan et al., 2008), enhanced problem‐solving skills (Drover et al., 2009; Judge et al., 2007), sustained attention (Colombo et al., 2016; Jensen et al., 2010), improved cognitive function (Birch et al., 2000; Drover et al., 2011; Willatts et al., 2016), enhanced gross motor milestones (Agostoni et al., 2009), and increased adjustability to environment (Gustafson et al., 2013). Judge et al. (2007) indicated that children whose mothers consumed cod liver oil with *n*‐3 LCPUFA (1,183 mg DHA and 803 mg EPA per day) from the 18th week of pregnancy until 3 months after the delivery scored higher on the mental processing composite of Kaufman Assessment Battery for Children (K‐ABC, intelligence testing) at the age of four than the control group. In a double‐blinded randomized controlled trial (RCT), which enrolled 181 infants at 1–9 days of age, the effects of DHA supplementation on mental delay of the infants were evaluated using Bayley Mental Delay Index (MDI; Drover et al., 2011). The results suggested that the infants who consumed the term infant formula containing DHA (between 0.32% and 0.96% of the total fatty acids) for 12 months obtained significantly higher MDI scores at the age of 18 months. A significant effect from the maternal intake of DHA was also reported in increasing the hand and eye coordination of toddlers at the age of 2.5 when the mothers utilized the DHA supplementation from the late gestation to the delivery (Dunstan et al., 2008). Similarly, the infants at the age of six fed with the formula enriched with DHA (0.2% of the total fatty acids) and arachidonic acid (ARA) for a period of 4 months showed a faster ability in processing information than the control group without the DHA and ARA supplementation (Willatts et al., 2016) although there were no significant differences in their intelligence quotients. It seems that both pregnancy and lactation are the critical periods to supplement DHA to infants in order to achieve the best neurodevelopmental effects of DHA (Helland et al., 2003).

### 2.2 Preterm birth

Preterm birth is a major problem of public health care, accounting for around 12% of the deliveries worldwide (Beck et al., 2010). It is an underlying risk factor for 75% of neonatal morbidity and various infant diseases (Lumley, 2003), generally due to the underdeveloped immune‐regulatory system and antigen‐specific adaptive immune system (Zhang et al., 2014).

A systematic review and meta‐analysis by Kar et al. (2016) found that *n*‐3 PUFA supplementation reduced the risk of preterm delivery (<37 weeks) by 17%. The mean gestational age at delivery in the *n*‐3 PUFA group was increased by 1.95 weeks compared with the control group. This may be attributed to the downregulating inflammatory effect of DHA by decreasing the contents of prostaglandin E_2_ and prostaglandin F_2α_ in the uterus (Roman et al., 2006). Similarly, Harris et al. (2015) demonstrated that the pregnant women supplemented with 300 mg algal DHA per day had a significantly lower risk of early preterm delivery than the control group without the DHA supplementation (1.7% vs. 5.7%). In addition, the group of pregnant women supplemented with 300 mg/day algal DHA carried their babies 4 days longer than the control group without the DHA supplementation and only 0.5 day shorter than the group with higher dose of the DHA supplementation (600 mg/day). Similar results of longer gestation period were observed from the studies employing fish oil to supplement pregnant women with DHA (Knudsen et al., 2006; Olsen et al., 2000) although the length of gestation period did not increase significantly when the dose of DHA exceeded 600 mg/day. These studies verified that the DHA intake during pregnancy can optimize the length of gestation period without the risks of delaying of the delivery date due to the extended gestation period.

### 2.3 Allergy and immunity

There are increasing evidences showing that women who take *n*‐3 LCPUFA during pregnancy and breastfeeding may protect their children against the development of allergies (Birch et al., 2010; Foiles et al., 2016; Furuhjelm et al., 2009; Hansen et al., 2016) due to the anti‐inflammatory mechanism of *n*‐3 LCPUFA. A systematic review and meta‐analysis involving 10 prospective cohort studies and five unique RCTs showed that the increase in the maternal *n*‐3 LCPUFA, such as by DHA supplementation, could reduce the offspring's risk of developing eczema (Best et al., 2016). In addition, a recent RCT (*n* = 533) with a 24‐year follow‐up revealed that *n*‐3 LCPUFA supplementation during the pregnancy stage could induce a prophylactic effect against the development of asthma in the offspring rather than provide a therapeutic effect (Hansen et al., 2016). It was reported that the probability of asthma discharge diagnosis was lower and less asthma medication was prescribed for the group supplemented with fish oil from childhood to early adulthood than the control group supplemented with olive oil. Similarly, there were less medical reports about the occurrence of allergic illnesses in the first 3 to 4 years of life for the children receiving formula containing DHA (0.32%–0.96% of total fatty acids) and ARA (0.64% of total fatty acids) than those without the DHA supplementation (Birch et al., 2010; Foiles et al., 2016). However, if the allergic immune responses have established, the intervention using DHA supplementation can be too late and will only show a small improvement (Thien et al., 2000).

### 2.4 Cardiovascular disease

CVD is the leading cause of mortality in the world. For example, the prevalence of CVD in China is still rising, and it was estimated that 330 million people were suffering from CVD in 2019 (The Writing Committee of the Report on Cardiovascular Health & Diseases in China, 2020). Some research studies have verified that *n*‐3 LCPUFA plays a role in the protection against atherosclerotic heart disease (Mori, 2014), sudden coronary death (Mozaffarian & Wu, 2011; Schmidt et al., 2005), and peripheral arterial disease (Dawczynski et al., 2010). In addition, *n*‐3 LCPUFA has multiple beneficial effects on reducing cardio‐metabolic risk factors, including dyslipidemia (Nestel et al., 2002), increased blood pressure (Geleijnse et al., 2002), arterial compliance (Nestel et al., 2002), vascular reactivity (Mori, Watts, et al., 2000), and inflammation (Ebrahimi et al., 2009; Mori, Burke, et al., 2000). A recent meta‐analysis conducted by Bernasconi et al. (2021) showed that there were 9% and 13% risk reductions in the coronary heart disease (CHD) events and myocardial infarction, respectively, with 1 g/day DHA supplements, and the effects were dose‐dependent.

In the past, the benefits of *n*‐3 LCPUFA in preventing CVD were studied using a mixture of EPA and DHA. In recent years, the individual effects of EPA and DHA on the risk factors of CVD have been revealed in order to have a better understanding of their mechanisms. Asztalos et al. (2016) recruited 121 healthy, normo‐lipidemic subjects, where the subjects were randomly assigned into four treatment groups: two doses of EPA (1,800 and 600 mg/day), one dose of DHA (600 mg/day), and olive oil without EPA and DHA (control). After 6 weeks of intervention, the DHA group showed a significant decrease in the postprandial TG concentration compared with the control group although the fasting TG remained unchanged. However, interestingly, the DHA group also showed a significant increase in the low‐density lipoprotein cholesterol (LDL) level. Although the EPA groups did not show the same results, there was a significant decrease in the lipoprotein‐associated phospholipase A_2_ concentration, which is an inflammatory marker, at the dose of 1,800 mg EPA/day. It seems that both EPA and DHA can decrease the CVD risk factors, and DHA may be more effective in reducing the lipid risk factors than EPA. This stronger effects of DHA in reducing fasting triglycerides were also reported by Grimsgaard et al. (1997) and Egert et al. (2009) from the healthy population using high doses of DHA (2.2 and 4 g/day, respectively), by Nestel et al. (2002) from the dyslipidemic patients, as well as by Allaire et al. (2016) and Mori, Burke, et al. (2000) from the obese subjects. Egert et al. (2009) also found that the HDL level was significantly increased by 21.2% in the DHA provision group.

Mori, Burke, et al. (2000), Allaire et al. (2016), and Bernstein et al. (2012) observed the increasing trends of the LDL level by the DHA supplementation either from fish oil or algal oil, confirming the finding of Asztalos et al. (2016). The increase in the LDL level could be attributed to the hypo‐triglyceridemic effect of DHA (Schmidt et al., 1993), where DHA supplementation decreases the TG level by reducing the hepatic synthesis of very low‐density lipoprotein (VLDL) and its secretion. This leads to the formation of small VLDL particles that are more readily to be converted to LDL than the large VLDL particles and thus increasing the LDL level (Mori, Burke, et al., 2000). In addition, the LDL particle size increased with the increased percentage of LDL when DHA supplementation was employed. The large LDL particle might have less atherogenic effects as the small and dense LDL particles are deemed to raise the risks of CVD (Campos et al., 1992).

### 2.5 Cognitive function

Cognitive function tends to reach the peak in middle age and then they gradually decline with age in healthy individuals (Hansen et al., 2016). Episodic memory typically declines after 20 years old, which coincides with the normal aging (Ellinson et al., 2004). Most observational studies (Beydoun et al., 2007; Muldoon et al., 2010; Titova et al., 2013) showed positive associations between DHA levels (in plasma, serum, or erythrocyte) and cognitive outcomes (such as working memory and composite cognitive scores) in healthy adults or healthy elderly.

A well‐designed meta‐analysis including 15 RCTs conducted by Yurko‐Mauro et al. (2015) demonstrated that DHA supplementation above the average of 580 mg/day significantly improved episodic memory outcomes in healthy adults (18 to 90 years old) with or without mild memory complaints and in the subgroup with mild memory complaints. However, it might need more than 4 months of intervention to obtain the benefits of DHA supplementation in improving cognitive decline. This can be reflected by the improvement in the semantic and work memory. For example, two trials, which supplemented subjects aged between 18 and 70 years old and between 18 and 35 years old with DHA at 850 mg and 1,000 mg/day, respectively, for 12 weeks (Jackson et al., 2012; Rogers et al., 2008), did not show any effects on cognition, whereas Stonehouse et al. (2013) reported an improvement in the episodic and working memory in 18‐ to 35‐year‐old subjects after an intervention with a high dose of DHA supplement (1,160 mg DHA and 170 mg EPA daily) for 6 months compared with the control subjects given the placebo treatment.

### 2.6 Eye health

Besides the need of DHA for the eye development of fetus and infant, some studies have shown that increased intake of *n*‐3 PUFA or fish consumption might lead to improve eye health, especially for elderly population. In some cohort studies (Christen et al., 2011; Chua et al., 2006) and a study involving twins with intermediate and late stages of age‐related macular degeneration (AMD; Seddon et al., 2006), their results showed that high intake of *n*‐3 PUFA and fish consumption might provide a protection against early and late AMD. There is still a need for further human studies in order to confirm the optimal DHA dose for the prevention of AMD. In addition, a review (Cortina & Bazan, 2011) has reported that the dietary supplementation with *n*‐3 PUFA showed some improvement in the signs and symptoms of dry eye, probably due to the anti‐inflammatory effect of DHA.

### 2.7 Prebiotics

The most recent widely accepted definition of prebiotics published by the International Scientific Association for Probiotics and Prebiotics or ISAPP (Gibson et al., 2017) is “a substrate that is selectively utilized by host microorganisms conferring a health benefit.” This new definition clarifies that the aims of prebiotics extend beyond the proliferation of bifidobacteria and lactobacilli and that the health benefits derived from these bacteria and other beneficial bacteria in the gut should be recognized.

Although the data are still limited to date, some studies have shown that DHA possesses prebiotic effects. A recent review (Fu et al., 2021) showed that *n*‐3 PUFA indirectly or directly modulate the gut microbiota, which leads to the reduction of pro‐inflammatory levels and the increase in short‐chain fatty acids. A randomized, open‐label, cross‐over trial providing 4 g/day of the combination of DHA and EPA (or 2 g DHA) in TG or EE form to healthy volunteers for 8 weeks reported that both DHA forms significantly increased the abundance of several beneficial genera (Watson et al., 2018), such as *Bifidobacterium*, *Roseburia*, and *Lactobacillus*, which are the main producers of butyrate in the gut. Butyrate is regarded as an important nutrient for the colonic mucosa, which in turn modulates gene expression, inflammation, differentiation, and apoptosis in host cells (Fu et al., 2021). Younge et al. (2017) observed that there were an increase in bacterial diversity and a decrease in the abundance of *Streptococcus*, *Clostridium*, and many pathogenic genera within the *Enterobacteriaceae* family in the infant group that was given high PUFA supplementation. The prebiotic effects of DHA, including the anti‐inflammatory properties, might be, at least in part, accountable for its other health benefits mentioned earlier, such as the prevention of CVD.

## 3 RECOMMENDED INTAKES OF DHA

Based on the growing body of evidences demonstrating the health benefits of DHA supplementation for different life stages, various expert bodies in different countries have made recommendations for the dietary intakes of DHA or *n*‐3 PUFA (as shown in Table 1). The RDI for DHA is in the range of 70 to 250 mg/day or 40 to 2,300 mg/day in combination with other *n*‐3 PUFA (Chinese Nutrition Society, 2013; Ezaki et al., 2012; Food & Agriculture Organization of the United Nations, 2010; Kris‐Etherton et al., 2002; National Health & Medical Research Council, 2006; Sposito et al., 2007; The French Food Safety Agency, 2010; Udell & Eden, 2008), depending on the age groups, genders, health conditions, and countries. In France, Japan, and Australia, there are detailed DHA recommendations for children based on the age groups.

**TABLE 1:** DHA recommendations in different countries^a^

Country/Age (year)	Japan (Ezaki et al., 2012)	France (The French Food Safety Agency, 2010)	FAO (Food & Agriculture Organization of the United Nations, 2010)	China (Chinese Nutrition Society, 2013)	WHO (Joint WHO/FAO Expert Consultation, 2003)	Australia (National Health & Medical Research Council, 2006	USA (Kris‐Etherton et al., 2002)
0–0.5	900 mg/day (*n*−3 PUFA)	0.32% of total fatty acids	0.1%–0.18% of total energy	100 mg/day		500 mg/day (*n*−3 PUFA)	
0.5–1	800 mg/day (*n*−3 PUFA)	70 mg/day		100 mg/day			
0.5–3	700–800 mg/day (*n*−3 PUFA)	70 mg/day		100 mg/day			
1–3		70 mg/day	100–150 mg/day (DHA and EPA)	100 mg/day		40 mg/day (*n*−3 PUFA)	700 mg/day (DHA and EPA)
3–4		125 mg/day		100 mg/day		55 mg/day (*n*−3 PUFA)	
3–9	1,100–1700 mg/day (*n*−3 PUFA)	125 mg/day	150–200 mg/day (DHA and EPA)			55 mg/day (*n*−3 PUFA)	
6–10			200–250 mg/day (DHA and EPA)			70 mg/day (*n*−3 PUFA)	
9–18	1,500–2,300 mg/day (*n*−3 PUFA)	250 mg/day				85 mg/day for girls 125 mg/day for boys (*n*−3 PUFA)	
General adult	≥2,000 mg/day (*n*−3 PUFA)	250 mg/day		250–2,000 mg/day (DHA and EPA)	1%–2% of total energy (*n*−3 PUFA)	500 mg/day (DHA and EPA; Udell & Eden, 2008)	500 mg/day (DHA and EPA)
Pregnant /lactating	1,800 mg/day (*n*−3 PUFA)	250 mg/day	300 mg/day (DHA and EPA)	200 mg/day		500 mg/day (DHA and EPA; Udell & Eden, 2008)	200 mg/day
CHD						1,000 mg/day (DHA and EPA; Udell & Eden, 2008)	1,000 mg/day (DHA and EPA)
CVD		500–750 mg/day (DHA and EPA)					

^a^ Unless it is specified, the recommendation is for DHA alone.

It was suggested by the Ministry of Health, Labour and Welfare of Japan (Ezaki et al., 2012) and National Health and Medical Research Council of Australia (2006) that the recommendation of *n*‐3 PUFA intake should be associated with the age groups and genders. For example, the recommendation for 0‐ to 5‐month‐old babies is 0.9 g total *n*‐3 PUFA per day, as the age increases, the recommendation increases to 2.0 and 2.4 g/day for women and men in the age of 50–69, respectively. In general, the RDI of total *n*‐3 PUFA for male in Japan is generally higher than that for female, except for infants at age of 0–2 years where there is no difference in the RDI for both genders. This can be related to the effects of hormones on the bioavailability of DHA, which is discussed in Section 5.4.

For the pregnant and lactating women, the RDI for DHA is around 200 mg in most countries, but Japan has a higher RDI (1,800 mg *n*‐3 PUFA) (Ezaki et al., 2012). In order to prevent CHD or CVD, the RDI for DHA and EPA can be increased to 1,000 mg for people at risk.

## 4 SOURCES OF DHA

Although DHA can be biosynthesized in the human body from α‐linolenic acid (ALA), which is abundant in some plants oils (e.g., flaxseed and perilla oils), under the actions of fatty acid elongases and desaturases, the bioconversion rate in the human body is extremely low, generally at 2%–10% (Chiu et al., 2008), and sometimes, it was reported even at a lower rate of 0.01% (Hussein et al., 2005). Therefore, DHA‐rich or fortified foods and DHA supplements are the two main exogenous sources to obtain additional DHA needed for the biological functions of human body.

### 4.1 Human breastmilk

Human breastmilk is the optimal and the most natural source of DHA for term infants. The average DHA concentration of human breastmilk is about 0.32 ± 0.22% of total fatty acids by weight worldwide (Brenna et al., 2007). There is a large variability that can be linked to the differences in the diets and the supplement intakes. For example, breastmilk with high DHA level is predominantly found in the seafood‐eating populations, such as Japan, Philippines, and Canadian Arctic (1.4%–0.6% DHA of total fatty acids; Innis et al., 1994; Yuhas et al., 2006), which are all coastal or island populations having plentiful seafood. In contrast, the lowest breastmilk DHA (0.06%–0.14% of total fatty acids) can be found in the developed or the inland countries, such as South Africa, United States, and Pakistan (Jensen et al., 2000; Smit et al., 2000; Van Der Westhuyzen et al., 1988). Hence, aside from providing nutrition education to mothers and expecting women, DHA supplement is a good way to achieve a higher DHA level in breastmilk. It has been shown that the DHA content in breastmilk is linearly correlated with the increase in the DHA supplementation (Sherry et al., 2015).

### 4.2 DHA‐rich foods

Seafood and its by‐products are known to contain substantial amount of *n*‐3 LCPUFA, including DHA. However, marine animals do not produce much *n*‐3 LCPUFA on their own. They accumulate *n*‐3 LCPUFA in their body by consuming PUFA‐producing marine microalgae and other smaller marine animals. Traditionally, cold‐water fish (primarily fatty fish) are recognized as the prominent diet sources of DHA, such as salmon, mackerel, herring, and trout. These fish can provide about 0.68–1.43 g DHA per 100 g meat. In addition, the meat, milk, and eggs of animals that are fed with seafood by‐products or PUFA‐producing microalgae have shown to contain DHA. The DHA contents of different foods are listed in Table 2.

**TABLE 2:** DHA contents of seafood, poultry, and livestock (Mozaffarian & Wu, 2012)

Food	DHA content (g/100 g meat)
Salmon, wild	1.43
Sardine, canned	1.2
Herring, Atlantic	1.1
Anchovy	1.3
Mackerel, Atlantic	0.70
Trout	0.68
Cod, Pacific	0.12
Shrimp	0.052
Prawn	0.04
Chicken breast	0.02
Pork	0.002

Based on the global survey on seafood consumption, the consumption level of 66% world adult population contains less than 250 mg *n*‐3 fat per day, lower than the recommended level (Marshall, 2011). This low consumption level can be associated with the geographical differences (coastland or inland), the living habits, and the concerns about environment protection and ocean contaminations (such as persistent organic pollutants, heavy mental, and methyl‐mercury).

### 4.3 DHA supplements

#### 4.3.1 *Fish* *oil*

Fish oil, as a dietary supplementation, offers an alternative means to the DHA‐rich foods in order to reach the recommended DHA level in the body. It is currently the most popular source for DHA supplements. Natural fish oils contain approximately 18% EPA and 12% DHA, which are mainly in TG form (Schuchardt & Hahn, 2013). Highly concentrated *n*‐3 PUFA oils containing up to 90% DHA and EPA in EE form have been developed for medical purposes, dietary supplements, and feedstock for various technological processes (such as for transesterification and re‐esterification). Furthermore, to produce a high quality, odorless, and stable fish oil, some additional processes, including distillation, purification, and deodorization, can be employed, but they can increase the production cost. In addition, there is a concern about the limitation of marine resources and the fishing quotas, decreasing the supply of fish oil and increasing its price. As reported by Salem and Eggersdorfer (2015), the DHA from fish oil can only meet 15% of human consumption demand in the world. Therefore, finding other sources for DHA supplements ​is crucial.

#### 4.3.2 *Krill* *oil*

As the amount of fish is limited, krill oil has surfaced to be an alternative source of marine *n*‐3 PUFA to fish oil. It is extracted from tiny shrimp‐like crustaceans, such as Antarctic krill (*Euphausia superba*), that feed on phytoplankton (including microalgae) in the deep ocean waters. Some of these phytoplankton synthesize large amounts of EPA and DHA. Krill oil contains both EPA and DHA similar to fish oil, albeit with lower contents of DHA and other *n*‐3 PUFA. However, it is still perceived as having higher efficacy than fish oil, probably, because 30%–65% of its fatty acids are in PL form (Tou et al., 2007; Ulven et al., 2011), which are easier to be absorbed in the human body than those in TG form; however, the results from current clinical studies are not yet sufficient to support the higher DHA bioavailability of krill oil (Salem & Kuratko, 2014), which is discussed in Section 5.1. In addition, similar to fish oil and other marine products, ecosystem sustainability and persistent organic pollutants are some of the concerns that the industry needs to address for krill oil.

#### 4.3.3 *Algal* *oil*

As mentioned in the previous sections, both fish and krill do not synthesize much DHA. They obtain most of their DHA through diets, including DHA‐producing microalgae, and store the DHA in their eyes and body lipids. There are many types of microalgae that can synthesize DHA, and *Schizochytrium* sp. is the most prominent microorganisms for *n*‐3 LCPUFA production by large‐scale fermentation technology. DHA‐rich oil from *Schizochytrium* sp. has been generally recognized as safety for food use and dietary supplement. The oil is primarily present in TG form and the commercial oil contains approximately 40% DHA of total fatty acids (Shahidi & Ambigaipalan, 2018). At present, although the market share of *n*‐3 LCPUFA supplement from *Schizochytrium* sp. is lower than that of marine animal oil, the growth is appreciable due to its vegetarian nature, environmental friendliness, nonexisting ocean pollutants, and compliance to expanding customer needs (such as kosher and halal). However, the fermentation and refining of algal oil currently cost more than the extraction and refining of fish oil.

## 5 DHA BIOAVAILABILITY

To exert the health benefits of DHA mentioned in Section 2, the bioavailability of DHA is a factor that cannot be neglected, apart from the DHA content of a food. Up to this moment, two reviews (Ghasemifard et al., 2014; Schuchardt & Hahn, 2013) have been published on the bioavailability of DHA, mainly focusing on the findings from human and animal studies as well as the methodological and analytical approaches to perform the studies. They concluded that there were still limitations in the previous studies on DHA bioavailability and there was lack of standardization of the analytical methods. Contrary to the clear definition of “bioavailability” in pharmacology, there is no agreed definition for nutrients, mainly due to their metabolisms and syntheses in the body (Kwan, 1997). The concentrations of DHA in free fatty acid (FFA), PL, and TG forms in plasma or serum have been used as indicators for DHA absorption rate and quantity to understand the short‐term bioavailability of DHA (Ghasemifard et al., 2014; Katan et al., 1997), while omega‐3 index has been regarded as a good biomarker for long‐term incorporation of *n*‐3 LCPUFA in the tissues (Harris & Von Schacky, 2004). Omega‐3 index is defined as the sum of EPA and DHA in the serum lipid fraction (cholesterol esters, triglycerides, and phospholipids) expressed as a percentage of the total fatty acids in erythrocyte membranes. It is also useful to discuss which factors influence the bioavailability of DHA and whether the higher bioavailability can be translated into a higher clinical efficacy. Different from pharmaceuticals, the bioavailability of DHA is highly dependent by numerous factors (Vandal et al., 2008), such as the physicochemical properties of the oil (including the chemical form and the position of *n*‐3 LCPUFA on the glycerol backbone), the matrix of the food or excipient (including the presence or absence of other food components), and the subjects (their state of health and their age).

### 5.1 Effects of chemical structure

The DHA oil from various sources can differ in terms of DHA contents and chemical forms or structures. In fish oil and algal oil, DHA is present primarily in TG form, where it is mainly bound on the sn‐2 position of the glycerol backbone and, to a less extent, in FFA form. Commercially available oils with high DHA content are normally in EE, FFA, and re‐esterified TG forms. DHA also naturally occurs in PL form, such as in krill oil.

Table 3 summarizes the experimental designs and outcomes of the published animal studies on DHA bioavailability. It can be inferred that the DHA bioavailability in EE form is lower than that in TG form (Yang et al., 1990) and monoglyceride (MG) form (Valenzuela et al., 2005) and that in FFA form has the highest bioavailability (Ikeda et al., 1995). Similar findings were reported for other *n*‐3 LCPUFA (Davidson et al., 2012; El Boustani et al., 1987; Lawson & Hughes, 1988b). The *n*‐3 LCPUFA in EE form might need an additional hydrolysis step to yield the DHA and/or EPA in FFA form before they can be incorporated into chylomicron for absorption. When the DHA in FFA form is absorbed into the blood stream or lymph, it will be re‐esterified into TG form. On the other hand, the TG form can provide glycerol and 2‐monoacylglyceride molecules that can speed up the re‐esterification process compared with the EE form, and hence, the delivery of DHA from enterocytes is not delayed (Kar et al., 2016). In addition, the *n*‐3 LCPUFA in EE form is up to 50 times more resistant to the hydrolysis by pancreatic lipase than that in natural TG form as shown in in vitro studies (Beckermann et al., 1990; Grimsgaard et al., 1998).

**TABLE 3:** Summary of published animal studies on DHA bioavailability

Animal model	Gender	Period of study (day)	Dose	Treatment	Bioavailability result	References
Wistar rats	Male	1	0.4 g lipid	Radioactive‐labeled FO (in TG form) and marine lipid liposome (PL form)	The absorption of the DHA in PL form is higher than the DHA in TG form	Cansell et al. (2003)
Sprague Dawley rats	Male	1	2 mg [^14^C] DHA/kg body weight	[^14^C]DHA‐PL, [^14^C]DHA‐TG, and a mixture of [^14^C]DHA‐TG and PL	The accumulation in brain is higher for the treatment with [^14^C]DHA‐PL than the other treatments in the old age group	Graf et al. (2010)
Rats	Male	1	1–3 ml oil or corresponding EE or methyl ester by stomach tube	MO and rapeseed oil (in TG, EE, and methyl ester forms)	The digestion and the absorption of the DHA in both EE and methyl ester forms were less than half of those of the DHA in TG form	Yang et al. (1990)
Mice		14–63	The sum of EPA and DHA varied from 0.12% to 3.07% of total diet	PL form and TG form	The concentration of DHA‐PL in the blood plasma was higher than that of DHA‐TG.	Rossmeisl et al. (2012)
Obese Zuker rats		28	0.5 g of EPA and DHA/100g of diet, equivalent to 0.8% of energy	FO and KO	The KO supplementation significantly increased the DHA in PL form than the FO supplementation	Batetta et al. (2009)
Obese Zuker rats		28	0.5 g EPA and DHA/100 g of diet, equivalent to 0.8% of energy	FO and KO	There was a higher DHA level in the brain PL of rats fed with KO than with FO	Di Marzo et al. (2010)
Long–Evans rats	Male	28	2% DHA of total fatty acids	[^3^H]PL‐DHA [^3^H]TG‐DHA	The rats fed with [^3^H] PL‐DHA had higher radioactivity in the brain.	Kitson et al. (2016)
				Olive oil (control), FO, PLC, and a mixture of FO and PLC	No differences were found among DHA‐supplemented groups for the DHA contents in brain and whole body as well as for the DHA accretion rates in tissues, but the blend of FO and PLC had higher DHA contents in the heart and serum than FO alone	
Wistar rats	Female	30	DHA 0.8mg/kg body weight	DHA‐MG, DHA‐EE, and Oleic acid EE (control)	The supplementation with DHA‐MG increased the DHA levels in plasma and erythrocyte more than that with DHA‐EE	Valenzuela et al. (2005)
Sprague Dawley rats	Female	56	12% of lipids in the diet	Corn oil (control), flaxseed oil, KO, MO, SO, and TO	The rats fed with KO had DHA level in the brain similar to those fed with corn oil and MO, which was significantly lower than those fed with SO and TO	Tou et al. (2011)

Abbreviations: FO, fish oil; KO, krill oil; MG, monoglycerides; MO, menhaden oil; PLC, herring caviar phospholipid concentrate; SO, salmon oil; TO, tuna oil.

Whether the DHA in PL form has a higher bioavailability than that in TG form is still not fully understood. Krill oil, rich in DHA with PL form, has been reported to have a higher absorption rate and a higher DHA accretion rate in plasma than fish oil, where the DHA is mainly in TG form (Batetta et al., 2009; Di Marzo et al., 2010; Ghasemifard et al., 2015; Graf et al., 2010; Kitson et al., 2016; Rossmeisl et al., 2012). In addition, Ulven et al. (2011) indirectly demonstrated that the bioavailability of DHA in krill oil may have higher efficacy than that in fish oil. They found no significant differences between krill oil and fish oil in the DHA levels of the plasma and serum lipids when the subjects were supplemented with the krill oil containing 53% of the DHA level in the fish oil used in the study. However, Tou et al. (2011) found that the DHA oil digestibility and the DHA accretion rate in the brain of the rats fed with krill oil were lower than those fed with salmon oil and tuna oil although the DHA content of the krill oil used in this study was 2 to 4 times higher than that in the other oils (4.9 mg/g diet vs. 1.9–2.9 mg/g diet). This could be attributed to the low proportion of PL in this particular krill oil, and the DHA was present in both PL and TG forms, where the PL form was only accounted for 50% of the total DHA. In another randomized study, there was no significant difference in the DHA level of red blood cell membrane between the TG and PL groups after 4 weeks of treatment when the amounts of DHA and EPA per treatment were maintained the same (Köhler et al., 2015). These inconsistent results could be attributed to the different contents of EPA and DHA, the doses, the duration of intervention, the different detection methods (such as the plasma DHA level, the whole blood fatty acids, and the omega‐3 index), the variations in krill oil compositions (19%–81% PL and 3.5%–36% FFA), and the various PL forms (e.g., phosphatidylethanolamine and phosphatidylserine). In the future, it might be worthy to explore the fatty acid compositions of plasma PL, red blood cells, and whole blood from a long‐term study using the equivalent dose of DHA from krill oil and fish oil.

### 5.2 Effect of food composition

It is well established that the composition of a food can affect the bioavailability of the nutrients (Parada & Aguilera, 2007), and thus, it can influence the bioavailability of *n*‐3 LCPUFA, as discussed by Schuchardt and Hahn (2013). Dietary lipids in meals have shown to significantly increase the bioavailability of DHA and EPA. Lawson and Hughes (1988a) found that the absorption of DHA and EPA from fish oil in EE from was threefold higher when the volunteers co‐ingested with a fat‐rich meal (44 g of fat) than those co‐ingested with a low‐fat meal (8 g of fat). This effect was more remarkable in the cross‐over study conducted by Kling et al. (2011). They demonstrated that the bioavailability of DHA and EPA in EE and FFA forms was increased significantly in the plasma during a high fat diet period compared with during a low‐fat diet period. For example, the *c*_max_ (the maximum concentration measured in the plasma over the specific timespan) and *t*_max_ (the time when the measured maximum concentration first occurred in the plasma) of total concentrations of DHA and EPA were five times higher and 0.6 time shorter, respectively, during high fat diet than those during low‐fat diet when the subjects were administrated with 4‐g dose of *n*‐3 LCPUFA in EE form. This positive effect might be due to the stimulating effect of dietary lipids on the secretion of pancreatic lipases into the small intestine. Similarly, the health benefits of DHA could be underestimated if the effect of food composition is neglected. For example, in one study performed in Germany (Rauch et al., 2010), no significant benefits of *n*‐3 LCPUFA in EE form were observed in reducing the CVD events when the subjects co‐ingested *n*‐3 LCPUFA with relatively low‐fat breakfast meal.

### 5.3 Effect of excipients

Some technologies, such as emulsion and microencapsulation, have been developed to mask the strong odor and to reduce the oxidation rate of PUFA oil. A large‐scale (*n* = 99), double‐blinded, randomized controlled, long‐term (4 weeks) study was conducted to compare the bioavailability of DHA and EPA between microencapsulated powder and enriched meals (Hinriksdottir et al., 2015). The results showed that there was no significant difference in the *n*‐3 LCPUFA levels between the intervention groups. A 2‐week study conducted by Arterburn et al. (2008) also found that algal DHA oil in soft‐gel capsules and cooked salmon with the same content of DHA (600 mg/day) had similar effects in increasing the DHA levels in plasma and red bloods cells. Therefore, DHA supplement is as effective as natural DHA sources.

Emulsified fish oil can significantly increase the bioavailability of EPA and DHA compared with the fish oil in gelatin‐based soft‐gel capsules (Haug et al., 2011; Raatz et al., 2009). Emulsion might improve the dissolution of the oil in the digestive tract for nutrient absorption, while the gelatin‐based capsules need to be broken down by pancreatic protease before the oil can be released for absorption, thus delaying the absorption time. However, there were some limitations of these studies. The sample sizes were very small (only 10 subjects), and the duration was only 48 hr. A larger sample size and a longer duration of study might be needed to verify these results.

### 5.4 Effects of hormones

Giltay et al. (2004) observed sex hormones played an opposite regulation of DHA content in the plasma: estrogen stimulated the biosynthesis of DHA, thus induced an increase in DHA status, whereas testosterone stimulus induced a decrease in DHA. The DHA concentrations in plasma cholesteryl esters in both female groups with or without taking oral contraceptives were significantly higher than those in the male group when they consumed the same amounts of DHA supplement. Similarly, in a recent review (Lohner et al., 2013), it was demonstrated that women had higher proportions of ARA and DHA in plasma, red blood cells, and adipose tissue lipids than men. This effect was also observed in a large cohort study of American children aged 6–16 years old. The cognitive benefits from consuming *n*‐3 PUFA were twice more prominent in girls than in boys (Lassek & Gaulin, 2011), which could be attributed to that estrogens from girls upregulated the DHA concentration in the plasma. It could also be the reason that the Japanese experts gave a higher RDI of *n*‐3 PUFA for men than that for women (seen in Section 3).

## 6 CONCLUSION

The *n*‐3 LCPUFA DHA is important throughout human lifespan and is a dietary necessity found predominantly in marine and algal oils. The consumption of DHA can provide many positive physiological and behavioral effects, including proper fetal development, prevention of premature delivery, prevention of infant allergy, improved cardiovascular functions in terms of anti‐inflammatory properties, and improved cognitive functions and eye health in adult and aging populations. These health benefits of DHA could be, at least in part, attributed to its prebiotic effects, which has been reported to modulate the gut microbiota. Apart from its sources, such as fish oil, krill oil, and algal oil, DHA can be found in different chemical forms including FFA, TG, PL, and EE. The EE form is not normally present in the nature, and it is employed to increase the concentration of DHA, mainly for pharmaceuticals and nutraceuticals. The bioavailability of DHA is also important to be considered and it is affected by some factors, such as the chemical form of DHA, food or excipient matrix, and hormones. Further study should be conducted to confirm the health benefits of DHA and the optimal recommendation intake of DHA for male and female at different age groups, similar to what the experts in Japan have recommended for DHA intakes. At present, the DHA intake in world is far below the recommendation intake, partly due to the limited marine resources and catching quotas as well as the concerns about the environmental pollution. DHA supplementation from algal oil or other sustainable alternatives is a good means to fulfill the increasing demands for DHA in the future.

## CONFLICT OF INTEREST

The authors also declare that they have no conflict of interest.

## AUTHOR CONTRIBUTIONS

**Jia Li:** Investigation (lead); Writing‐original draft (lead). **Bernard L. R. Pora:** Conceptualization (lead); Supervision (supporting). **Ke Dong:** Supervision (supporting); Writing‐review & editing (supporting). **Jovin Hasjim:** Supervision (lead); Writing‐review & editing (lead).

## Data Availability

Data sharing not applicable to this review as no datasets were generated from this review.

## References

[fsn32299-bib-0001] Agostoni, C., Zuccotti, G. V., Radaelli, G., Besana, R., Podestà, A., Sterpa, A., Rottoli, A., Riva, E., & Giovannini, M. (2009). Docosahexaenoic acid supplementation and time at achievement of gross motor milestones in healthy infants: A randomized, prospective, double‐blind, placebo‐controlled trial. The American Journal of Clinical Nutrition, 89(1), 64–70. 10.3945/ajcn.2008.26590 19056592

[fsn32299-bib-0002] Allaire, J., Couture, P., Leclerc, M., Charest, A., Marin, J., Lépine, M. C., Talbot, D., Tchernof, A., & Lamarche, B. (2016). A randomized, crossover, head‐to‐head comparison of eicosapentaenoic acid and docosahexaenoic acid supplementation to reduce inflammation markers in men and women: The comparing EPA to DHA (ComparED) study. The American Journal of Clinical Nutrition, 104(2), 280–287. 10.3945/AJCN.116.131896 27281302

[fsn32299-bib-0003] Arterburn, L. M., Oken, H. A., Bailey Hall, E., Hamersley, J., Kuratko, C. N., & Hoffman, J. P. (2008). Algal‐oil capsules and cooked salmon: Nutritionally equivalent sources of Docosahexaenoic acid. Journal of the American Dietetic Association, 108(7), 1204–1209. 10.1016/j.jada.2008.04.020 18589030

[fsn32299-bib-0004] Asztalos, I. B., Gleason, J. A., Sever, S., Gedik, R., Asztalos, B. F., Horvath, K. V., Dansinger, M. L., Lamon‐Fava, S., & Schaefer, E. J. (2016). Effects of eicosapentaenoic acid and docosahexaenoic acid on cardiovascular disease risk factors: A randomized clinical trial. Metabolism, 65(11), 1636–1645. 10.1016/j.metabol.2016.07.010 27733252

[fsn32299-bib-0005] Batetta, B., Griinari, M., Carta, G., Murru, E., Ligresti, A., Cordeddu, L., Giordano, E., Sanna, F., Bisogno, T., Uda, S., Collu, M., Bruheim, I., Di Marzo, V., & Banni, S. (2009). Endocannabinoids may mediate the ability of *n*‐3 fatty acids to reduce ectopic fat and inflammatory mediators in obese Zucker rats. The Journal of Nutrition, 139(8), 1495–1501. 10.3945/jn.109.104844 19549757

[fsn32299-bib-0006] Beck, S., Wojdyla, D., Say, L., Betran, A. P., Merialdi, M., Requejo, J. H., Rubens, C., Menon, R., & Van Look, P. F. A. (2010). The worldwide incidence of preterm birth: A systematic review of maternal mortality and morbidity. Bulletin of the World Health Organization, 88(1), 31–38. 10.2471/BLT.08.062554 20428351 PMC2802437

[fsn32299-bib-0007] Beckermann, B., Beneke, M., & Seitz, I. (1990). Comparative bioavailability of eicosapentaenoic acid and docosahexaenoic acid from triglycerides, free fatty acids and ethyl esters in volunteers. Arzneimittel‐Forschung, 40, 700–704. 2144420

[fsn32299-bib-0008] Bernasconi, A. A., Wiest, M. M., Lavie, C. J., Milani, R. V., & Laukkanen, J. A. (2021). Effect of Omega‐3 dosage on cardiovascular outcomes: An updated meta‐analysis and meta‐regression of interventional trials. Mayo Clinic Proceedings, 96(2), 304–313. 10.1016/j.mayocp.2020.08.034 32951855

[fsn32299-bib-0009] Bernstein, A. M., Ding, E. L., Willett, W. C., & Rimm, E. B. (2012). A meta‐analysis shows that docosahexaenoic acid from algal oil reduces serum triglycerides and increases HDL‐cholesterol and LDL‐cholesterol in persons without coronary heart disease. The Journal of Nutrition, 142(1), 99–104. 10.3945/jn.111.148973 22113870

[fsn32299-bib-0010] Best, K. P., Gold, M., Kennedy, D., Martin, J., & Makrides, M. (2016). Omega‐3 long‐chain PUFA intake during pregnancy and allergic disease outcomes in the offspring: A systematic review and meta‐analysis of observational studies and randomized controlled trials. The American Journal of Clinical Nutrition, 103, 128–143. 10.3945/ajcn.115.111104.1 26675770

[fsn32299-bib-0011] Beydoun, M. A., Kaufman, J. S., Satia, J. A., Rosamond, W., & Folsom, A. R. (2007). Plasma *n*‐3 fatty acids and the risk of cognitive decline in older adults: The atherosclerosis risk in communities study. American Journal of Clinical Nutrition, 85(4), 1103–1111. 10.1093/ajcn/85.4.1103 17413112

[fsn32299-bib-0012] Birch, E. E., Garfield, S., Hoffman, D. R., Uauy, R., & Birch, D. G. (2000). A randomized controlled trial of early dietary supply of long‐chain polyunsaturated fatty acids and mental development in term infants. Developmental Medicine and Child Neurology, 42(3), 174–181. 10.1017/S0012162200000311 10755457

[fsn32299-bib-0013] Birch, E. E., Khoury, J. C., Berseth, C. L., Castañeda, Y. S., Couch, J. M., Bean, J., Tamer, R., Harris, C. L., Mitmesser, S. H., & Scalabrin, D. M. (2010). The impact of early nutrition on incidence of allergic manifestations and common respiratory illnesses in children. Journal of Pediatrics, 156(6), 902–906.e1. 10.1016/j.jpeds.2010.01.002 20227721

[fsn32299-bib-0014] Brenna, J. T., Varamini, B., Jensen, R. G., Diersen‐Schade, D. A., Boettcher, J. A., & Arterburn, L. M. (2007). Docosahexaenoic and arachidonic acid concentrations in human breast milk worldwide. American Journal of Clinical Nutrition, 85(6), 1457–1464. 10.1093/ajcn/85.6.1457 17556680

[fsn32299-bib-0015] Campos, H., Genest, J. J., Blijlevens, E., McNamara, J. R., Jenner, J. L., Ordovas, J. M., Wilson, P. W., & Schaefer, E. J. (1992). Low density lipoprotein particle size and coronary artery disease. Arteriosclerosis and Thrombosis: A Journal of Vascular Biology, 12(2), 187–195. 10.1161/01.atv.12.2.187 1543692

[fsn32299-bib-0016] Cansell, M., Nacka, F., & Combe, N. (2003). Marine lipid‐based liposomes increase in vivo FA bioavailability. Lipids, 38(5), 551–559. 10.1007/s11745-003-1341-0 12880112

[fsn32299-bib-0017] Carver, J. D., Benford, V. J., Han, B., & Cantor, A. B. (2001). The relationship between age and the fatty acid composition of cerebral cortex and erythrocytes in human subjects. Brain Research Bulletin, 56(2), 79–85. 10.1016/S0361-9230(01)00551-2 11704343

[fsn32299-bib-0018] Chinese Nutrition Society . (2013). Chinese dietary reference intakes. China Science Publishing and Media Ltd.

[fsn32299-bib-0019] Chiu, C. C., Su, K. P., Cheng, T. C., Liu, H. C., Chang, C. J., Dewey, M. E., Stewart, R., & Huang, S. Y. (2008). The effects of omega‐3 fatty acids monotherapy in Alzheimer’s disease and mild cognitive impairment: A preliminary randomized double‐blind placebo‐controlled study. Progress in Neuro‐Psychopharmacology & Biological Psychiatry, 32(6), 1538–1544. 10.1016/j.pnpbp.2008.05.015 18573585

[fsn32299-bib-0020] Christen, W. G., Schaumberg, D. A., Glynn, R. J., & Buring, J. E. (2011). Dietary ω‐3 fatty acid and fish intake and incident age‐related macular degeneration in women. Archives of Ophthalmology, 129(7), 921–929. 10.1001/archophthalmol.2011.34 21402976 PMC3134638

[fsn32299-bib-0021] Chua, B., Flood, V. M., Rochtchina, E., Wang, J. J., Smith, W., & Mitchell, P. (2006). Dietary fatty acids and the 5‐year incidence of age‐related maculopathy. Archives of Ophthalmology, 124(7), 981–986. 10.1001/archopht.124.7.981 16832021

[fsn32299-bib-0022] Colombo, J., Gustafson, K. M., Gajewski, B. J., Shaddy, D. J., Kerling, E. H., Thodosoff, J. M., Doty, T., Brez, C. C., & Carlson, S. E. (2016). Prenatal DHA supplementation and infant attention. Pediatric Research, 80(5), 656–662. 10.1038/pr.2016.134 27362506 PMC5164926

[fsn32299-bib-0023] Cortina, M. S., & Bazan, H. E. (2011). Docosahexaenoic acid, protectins and dry eye. Current Opinion in Clinical Nutrition and Metabolic Care, 14(2), 132–137. 10.1097/mco.0b013e328342bb1a 21157308 PMC3971926

[fsn32299-bib-0024] Davidson, M. H., Johnson, J., Rooney, M. W., Kyle, M. L., & Kling, D. F. (2012). A novel omega‐3 free fatty acid formulation has dramatically improved bioavailability during a low‐fat diet compared with omega‐3‐acid ethyl esters: The ECLIPSE (Epanova® compared to Lovaza^®^ in a pharmacokinetic single‐dose evaluation) study. Journal of Clinical Lipidology, 6(6), 573–584. 10.1016/j.jacl.2012.01.002 23312053

[fsn32299-bib-0025] Dawczynski, C., Martin, L., Wagner, A., & Jahreis, G. (2010). *N*‐3 LC‐PUFA enriched dairy products are able to reduce cardiovascular risk factors: A double‐blind, cross‐over study. Clinical Nutrition, 29(5), 592–599. 10.1016/j.clnu.2010.02.008 20304540

[fsn32299-bib-0026] Di Marzo, V., Griinari, M., Carta, G., Murru, E., Ligresti, A., Cordeddu, L., Giordano, E., Bisogno, T., Collu, M., Batetta, B., Uda, S., Berge, K., & Banni, S. (2010). Dietary krill oil increases docosahexaenoic acid and reduces 2‐arachidonoylglycerol but not N‐acylethanolamine levels in the brain of obese Zucker rats. International Dairy Journal, 20(4), 231–235. 10.1016/j.idairyj.2009.11.015

[fsn32299-bib-0027] Drover, J. R., Hoffman, D. R., Castañeda, Y. S., Morale, S. E., & Birch, E. E. (2009). Three randomized controlled trials of early long‐chain polyunsaturated fatty acid supplementation on means‐end problem solving in 9‐month‐olds. Child Development, 80(5), 1376–1384. 10.1111/j.1467-8624.2009.01339.x 19765006 PMC2757317

[fsn32299-bib-0028] Drover, J. R., Hoffman, D. R., Castañeda, Y. S., Morale, S. E., Garfield, S., Wheaton, D. H., & Birch, E. E. (2011). Cognitive function in 18‐month‐old term infants of the DIAMOND study: A randomized, controlled clinical trial with multiple dietary levels of docosahexaenoic acid. Early Human Development, 87(3), 223–230. 10.1016/j.earlhumdev.2010.12.047 21295417

[fsn32299-bib-0029] Dunstan, J. A., Simmer, K., Dixon, G., & Prescott, S. L. (2008). Cognitive assessment of children at age 2(1/2) years after maternal fish oil supplementation in pregnancy: A randomised controlled trial. Archives of Disease in Childhood. Fetal and Neonatal Edition, 93(1), F45–F50. 10.1136/adc.2006.099085 17185423

[fsn32299-bib-0030] Ebrahimi, M., Ghayour‐Mobarhan, M., Rezaiean, S., Hoseini, M., Parizade, S. M. R., Farhoudi, F., Hosseininezhad, S. J., Tavallaei, S., Vejdani, A., Azimi‐Nezhad, M., Shakeri, M. T., Rad, M. A., Mobarra, N., Kazemi‐Bajestani, S. M. R., & Ferns, G. A. A. (2009). Omega‐3 fatty acid supplements improve the cardiovascular risk profile of subjects with metabolic syndrome, including markers of inflammation and auto‐immunity. Acta Cardiologica, 64(3), 321–327. 10.2143/AC.64.3.2038016 19593941

[fsn32299-bib-0031] Egert, S., Kannenberg, F., Somoza, V., Erbersdobler, H. F., & Wahrburg, U. (2009). Dietary α‐linolenic acid, EPA, and DHA have differential effects on LDL fatty acid composition but similar effects on serum lipid profiles in normolipidemic humans. The Journal of Nutrition, 139(5), 861–868. 10.3945/jn.108.103861 19261730

[fsn32299-bib-0032] El Boustani, S., Colette, C., Monnier, L., Descomps, B., Crastes de Paulet, A., & Mendy, F. (1987). Enteral absorption in man of eicosapentaenoic acid in different chemical forms. Lipids, 22(10), 711–714. 10.1007/BF02533970 2828810

[fsn32299-bib-0033] Ellinson, M., Thomas, J., & Patterson, A. (2004). A critical evaluation of the relationship between serum vitamin B12, folate and total homocysteine with cognitive impairment in the elderly. Journal of Human Nutrition and Dietetics, 17(4), 371–383. 10.1111/j.1365-277X.2004.00532.x 15250847

[fsn32299-bib-0034] Ezaki, O., Miyake, Y., Sato, S., & Iso, H. (2012). Dietary reference intakes for Japanese 2010: Fat. Journal of Nutritional Science and Vitaminology, 59(Supplement), S44–S52. 10.3177/jnsv.59.S44

[fsn32299-bib-0035] Foiles, A. M., Kerling, E. H., Wick, J. A., Scalabrin, D. M. F., Colombo, J., & Carlson, S. E. (2016). Formula with long‐chain polyunsaturated fatty acids reduces incidence of allergy in early childhood. Pediatric Allergy and Immunology, 27(2), 156–161. 10.1111/pai.12515 26613373 PMC5207026

[fsn32299-bib-0036] Food and Agriculture Organization of the United Nations . (2010). Fats and fatty acids in human nutrition. Report of an expert consultation. FAO Food and Nutrition Paper, 91, 1–166. http://www.fao.org/docrep/013/i1953e/i1953e00.pdf 21812367

[fsn32299-bib-0037] Fu, Y. W., Wang, Y. D., Gao, H., Li, D. H., Jiang, R. R., Ge, L. R., Tong, C., & Xu, K. (2021). Associations among dietary omega‐3 polyunsaturated fatty acids, the gut microbiota, and intestinal immunity. Mediators of Inflammation, 2021, 11. 10.1155/2021/8879227 PMC7801035 33488295

[fsn32299-bib-0038] Furuhjelm, C., Warstedt, K., Larsson, J., Fredriksson, M., Böttcher, M. F., Fälth‐Magnusson, K., & Duchén, K. (2009). Fish oil supplementation in pregnancy and lactation may decrease the risk of infant allergy. Acta Paediatrica, International Journal of Paediatrics, 98(9), 1461–1467. 10.1111/j.1651-2227.2009.01355.x 19489765

[fsn32299-bib-0039] Geleijnse, J. M., Giltay, E. J., Grobbee, D. E., Donders, A. R. T., & Kok, F. J. (2002). Blood pressure response to fish oil supplementation: Metaregression analysis of randomized trials. Journal of Hypertension, 20(8), 1493–1499. 10.1097/00004872-200208000-00010 12172309

[fsn32299-bib-0040] Ghasemifard, S., Hermon, K., Turchini, G. M., & Sinclair, A. J. (2015). Metabolic fate (absorption, β‐oxidation and deposition) of long‐chain *n*‐3 fatty acids is affected by sex and by the oil source (krill oil or fish oil) in the rat. British Journal of Nutrition, 114(5), 684–692. 10.1017/S0007114515002457 26234617

[fsn32299-bib-0041] Ghasemifard, S., Turchini, G. M., & Sinclair, A. J. (2014). Omega‐3 long chain fatty acid “bioavailability”: A review of evidence and methodological considerations. Progress in Lipid Research, 56, 92–108. 10.1016/j.plipres.2014.09.001 25218856

[fsn32299-bib-0042] Gibson, G. R., Hutkins, R., Sanders, M. E., Prescott, S. L., Reimer, R. A., Salminen, S. J., Scott, K., Stanton, C., Swanson, K. S., Cani, P. D., Verbeke, K., & Reid, G. (2017). Expert consensus document: The international scientific association for probiotics and prebiotics (ISAPP) consensus statement on the definition and scope of prebiotics. Nature Reviews Gastroenterology and Hepatology, 14(8), 491–502. 10.1038/nrgastro.2017.75 28611480

[fsn32299-bib-0043] Giltay, E. J., Gooren, L. J. G., Toorians, A. W. F. T., Katan, M. B., & Zock, P. L. (2004). Docosahexaenoic acid concentrations are higher in women than in men because of estrogenic effects. The American Journal of Clinical Nutrition, 80(5), 1167–1174. 10.1093/ajcn/80.5.1167 15531662

[fsn32299-bib-0044] Graf, B. A., Duchateau, G. S. M. J. E., Patterson, A. B., Mitchell, E. S., van Bruggen, P., Koek, J. H., Melville, S., & Verkade, H. J. (2010). Age dependent incorporation of 14C‐DHA into rat brain and body tissues after dosing various 14C‐DHA‐esters. Prostaglandins Leukotrienes and Essential Fatty Acids, 83(2), 89–96. 10.1016/j.plefa.2010.05.004 20580213

[fsn32299-bib-0045] Grantham‐McGregor, S., Cheung, Y. B., Cueto, S., Glewwe, P., Richter, L., & Strupp, B. (2007). Developmental potential in the first 5 years for children in developing countries. The Lancet, 369(9555), 60–70. 10.1016/S0140-6736(07)60032-4 PMC2270351 17208643

[fsn32299-bib-0046] Grimsgaard, S., Bønaa, K. H., Hansen, J. B., & Myhre, E. S. (1998). Effects of highly purified eicosapentaenoic acid and docosahexaenoic acid on hemodynamics in humans. The American Journal of Clinical Nutrition, 68(1), 52–59. 10.1007/s11745-998-0188-8 9665096

[fsn32299-bib-0047] Grimsgaard, S., Bonaa, K. H., Hansen, J. B., & Nordøy, A. (1997). Highly purified eicosapentaenoic acid and docosahexaenoic acid in humans have similar triacylglycerol‐lowering effects but divergent effects on serum fatty acids. American Journal of Clinical Nutrition, 66(3), 649–659. 10.1093/ajcn/66.3.649 9280188

[fsn32299-bib-0048] Gustafson, K. M., Carlson, S. E., Colombo, J., Yeh, H.‐W., Shaddy, D. J., Li, S., & Kerling, E. H. (2013). Effects of docosahexaenoic acid supplementation during pregnancy on fetal heart rate and variability: A randomized clinical trial. Prostaglandins, Leukotrienes, and Essential Fatty Acids, 88(5), 331–338. 10.1016/j.plefa.2013.01.009 PMC3734850 23433688

[fsn32299-bib-0049] Haag, M. (2003). Essential fatty acids and the brain. Canadian Journal of Psychiatry, 48(3), 195–203. 10.1177/070674370304800308 12728744

[fsn32299-bib-0050] Hansen, S., Strom, M., Maslova, E., Dahl, R., Hoffmann, H. J., Rytter, D., Bech, B. H., Henriksen, T. B., Granstrom, C., Halldorsson, T. I., Chavarro, J. E., Linneberg, A., & Olsen, S. F. (2016). Fish oil supplementation during pregnancy and allergic respiratory disease in the adult offspring. The Journal of Allergy and Clinical Immunology, 139(1), 104–111. 10.1016/j.jaci.2016.02.042 27246522

[fsn32299-bib-0051] Harris, M. A., Reece, M. S., McGregor, J. A., Wilson, J. W., Burke, S. M., Wheeler, M., Anderson, J. E., Auld, G. W., French, J. I., & Allen, K. G. D. (2015). The effect of omega‐3 docosahexaenoic Acid supplementation on gestational length: Randomized trial of supplementation compared to nutrition education for increasing *n*‐3 intake from foods. BioMed Research International, 2015(6), 1–8. 10.1155/2015/123078 PMC4564584 26413500

[fsn32299-bib-0052] Harris, W. S., & Von Schacky, C. (2004). The Omega‐3 Index: A new risk factor for death from coronary heart disease? Preventive Medicine, 39(1), 212–220. 10.1016/j.ypmed.2004.02.030 15208005

[fsn32299-bib-0053] Haug, I. J., Sagmo, L. B., Zeiss, D., Olsen, I. C., Draget, K. I., & Seternes, T. (2011). Bioavailability of EPA and DHA delivered by gelled emulsions and soft gel capsules. European Journal of Lipid Science and Technology, 113(2), 137–145. 10.1002/ejlt.201000450

[fsn32299-bib-0054] Helland, I. B., Smith, L., Saarem, K., Saugstad, O. D., & Drevon, C. A. (2003). Maternal supplementation with very‐long‐chain *n*‐3 fatty acids during pregnancy and lactation augments children’s IQ at 4 years of age. Pediatrics, 111(1), 39–44. 10.1542/peds.111.1.e39 12509593

[fsn32299-bib-0055] Hinriksdottir, H. H., Jonsdottir, V. L., Sveinsdottir, K., Martinsdottir, E., & Ramel, A. (2015). Bioavailability of long‐chain *n*‐3 fatty acids from enriched meals and from microencapsulated powder. European Journal of Clinical Nutrition, 69(4), 344–348. 10.1038/ejcn.2014.250 25406967

[fsn32299-bib-0056] Hussein, N., Ah‐Sing, E., Wilkinson, P., Leach, C., Griffin, B., & Millward, D. J. (2005). Long‐chain conversion of [13C] linoleic acid and α‐linolenic acid in response to marked changes in their dietary intake in men. Journal of Lipid Research, 46(2), 269–280. 10.1194/jlr.M400225-JLR200 15576848

[fsn32299-bib-0057] Ikeda, I., Sasaki, E., Yasunami, H., Nomiyama, S., Nakayama, M., Sugano, M., Imaizumi, K., & Yazawa, K. (1995). Digestion and lymphatic transport of eicosapentaenoic and docosahexaenoic acids given in the form of triacylglycerol, free acid and ethyl ester in rats. Biochimica Et Biophysica Acta, 1259(3), 297–304. 10.1016/0005-2760(95)00180-8 8541338

[fsn32299-bib-0058] Innis, S. M., Nelson, C. M., Rioux, M. F., & King, D. J. (1994). Development of visual acuity in relation to plasma and erythrocyte ω‐6 and ω‐3 fatty acids in healthy term gestation infants. American Journal of Clinical Nutrition, 60(3), 347–352. 10.1093/ajcn/60.3.347 8074064

[fsn32299-bib-0059] Jackson, P. A., Deary, M. E., Reay, J. L., Scholey, A. B., & Kennedy, D. O. (2012). No effect of 12 weeks’ supplementation with 1 g DHA‐rich or EPA‐rich fish oil on cognitive function or mood in healthy young adults aged 18–35 years. British Journal of Nutrition, 107(08), 1232–1243. 10.1017/S000711451100403X 21864417

[fsn32299-bib-0060] Jensen, C. L., Maude, M., Anderson, R. E., & Heird, W. C. (2000). Effect of docosahexaenoic acid supplementation of lactating women on the fatty acid composition of breast milk lipids and maternal and infant plasma phospholipids. American Journal of Clinical Nutrition, 71(1 Suppl.), 292S–S299. 10.1093/ajcn/71.1.292s 10617985

[fsn32299-bib-0061] Jensen, C. L., Voigt, R. G., Llorente, A. M., Peters, S. U., Prager, T. C., Zou, Y. L., Rozelle, J. C., Turcich, M. R., Fraley, J. K., Anderson, R. E., & Heird, W. C. (2010). Effects of early maternal docosahexaenoic acid intake on neuropsychological status and visual acuity at five years of age of breast‐fed term infants. Journal of Pediatrics, 157(6), 900–905. 10.1016/j.jpeds.2010.06.006 20655543

[fsn32299-bib-0062] Joint WHO/FAO Expert Consultation . (2003). Diet, nutrition and the prevention of chronic diseases: Report of a joint WHO/FAO expert consultation. WHO technical report series 916. World Health Organization. doi: 10.1093/ajcn/60.4.644a 12768890

[fsn32299-bib-0063] Judge, M. P., Harel, O., & Lammi‐Keefe, C. J. (2007). Maternal consumption of a docosahexaenoic acid‐containing functional food during pregnancy: Benefit for infant performance on problem‐solving but not on recognition memory tasks at age 9 mo. American Journal of Clinical Nutrition, 85(6), 1572–1577. 10.1016/S1090-798X(08)79208-7 17556695

[fsn32299-bib-0064] Kar, S., Wong, M., Rogozinska, E., & Thangaratinam, S. (2016). Effects of omega‐3 fatty acids in prevention of early preterm delivery: A systematic review and meta‐analysis of randomized studies. European Journal of Obstetrics and Gynecology and Reproductive Biology, 198, 40–46. 10.1016/j.ejogrb.2015.11.033 26773247

[fsn32299-bib-0065] Katan, M. B., Deslypere, J. P., Van Birgelen, A. P. J. M., Penders, M., & Zegwaard, M. (1997). Kinetics of the incorporation of dietary fatty acids into serum cholesteryl esters, erythrocyte membranes, and adipose tissue: An 18‐month controlled study. Journal of Lipid Research, 38(10), 2012–2022. 10.1016/s0022-2275(20)37132-7 9374124

[fsn32299-bib-0066] Kidd, P. M. (2007). Omega‐3 DHA and EPA for cognition, behavior, and mood: Clinical findings and structural‐functional synergies with cell membrane phospholipids. Alternative Medicine Review, 12(3), 207–227. 10.1155/2013/824563 18072818

[fsn32299-bib-0067] Kitson, A. P., Metherel, A. H., Chen, C. T., Domenichiello, A. F., Trepanier, M. O., Berger, A., & Bazinet, R. P. (2016). Effect of dietary docosahexaenoic acid (DHA) in phospholipids or triglycerides on brain DHA uptake and accretion. Journal of Nutritional Biochemistry, 33, 91–102. 10.1016/j.jnutbio.2016.02.009 27135386

[fsn32299-bib-0068] Kling, D. F., Johnson, J., Rooney, M., & Davidson, M. H. (2011). Omega‐3 free fatty acids demonstrate more than 4‐fold greater bioavailability for EPA and DHA compared with omega‐3‐acid ethyl esters in conjunction with a low‐fat diet: The ECLIPSE study. Journal of Clinical Lipidology, 5(3), 231. 10.1016/j.jacl.2011.03.062 23312053

[fsn32299-bib-0069] Knudsen, V. K., Hansen, H. S., Osterdal, M. L., Mikkelsen, T. B., Mu, H., & Olsen, S. F. (2006). Fish oil in various doses or flax oil in pregnancy and timing of spontaneous delivery: A randomised controlled trial. Obstetrical & Gynecological Survey, 61(10), 622–623. 10.1097/01.ogx.0000238608.98535.ed 16579802

[fsn32299-bib-0070] Köhler, A., Sarkkinen, E., Tapola, N., Niskanen, T., & Bruheim, I. (2015). Bioavailability of fatty acids from krill oil, krill meal and fish oil in healthy subjects—A randomized, single‐dose, cross‐over trial. Lipids in Health and Disease, 14, 19. 10.1186/s12944-015-0015-4 25884846 PMC4374210

[fsn32299-bib-0071] Kris‐Etherton, P. M., Harris, W. S., & Appel, L. J. (2002). Fish consumption, fish oil, omega‐3 fatty acids, and cardiovascular disease. Circulation, 106(21), 2747–2757. 10.1161/01.CIR.0000038493.65177.94 12438303

[fsn32299-bib-0072] Kwan, K. C. (1997). Oral bioavailability and first‐pass effects. Drug Metabolism and Disposition, 25(12), 1329–1336. 9394021

[fsn32299-bib-0073] Lassek, W. D., & Gaulin, S. J. C. (2011). Sex differences in the relationship of dietary fatty acids to cognitive measures in American children. Frontiers in Evolutionary Neuroscience, 3, 1–8. 10.3389/fnevo.2011.00005 22065957 PMC3206402

[fsn32299-bib-0074] Lauritzen, L., Hansen, H. S., Jørgensen, M. H., & Michaelsen, K. F. (2001). The essentiality of long chain *n*‐3 fatty acids in relation to development and function of the brain and retina. Progress in Lipid Research, 40(1–2), 1–94. 10.1016/S0163-7827(00)00017-5 11137568

[fsn32299-bib-0075] Lawson, L. D., & Hughes, B. G. (1988a). Absorption of eicosapentaenoic acid and docosahexaenoic acid from fish oil triacylglycerols or fish oil ethyl esters co‐ingested with a high‐fat meal. Biochemical and Biophysical Research Communications, 156(2), 960–963. 10.1016/S0006-291X(88)80937-9 2847723

[fsn32299-bib-0076] Lawson, L. D., & Hughes, B. G. (1988b). Human absorption of fish oil fatty acids as triacylglycerols, free acids, or ethyl esters. Biochemical and Biophysical Research Communications, 152(1), 328–335. 10.1016/S0006-291X(88)80718-6 3358766

[fsn32299-bib-0077] Lohner, S., Fekete, K., Marosvölgyi, T., & Decsi, T. (2013). Gender differences in the long‐chain polyunsaturated fatty acid status: Systematic review of 51 publications. Annals of Nutrition & Metabolism, 62(2), 98–112. 10.1159/000345599 23327902

[fsn32299-bib-0078] Lumley, J. (2003). Defining the problem: The epidemiology of preterm birth. British Journal of Obstetrics and Gynaecology, 110(Suppl. 20), 3–7. 10.1046/j.1471-0528.2003.00011.x 12763104

[fsn32299-bib-0079] Marshall, T. A. (2011). Dietary guidelines for Americans, 2010: An update. Advances in Nutrition, 142(6), 654–656. 10.14219/jada.archive.2011.0248 21628687

[fsn32299-bib-0080] Micha, R., Khatibzadeh, S., Shi, P., Fahimi, S., Lim, S., Andrews, K. G., Engell, R. E., Powles, J., Ezzati, M., & Mozaffarian, D. (2014). Global, regional, and national consumption levels of dietary fats and oils in 1990 and 2010: A systematic analysis including 266 country‐specific nutrition surveys. BMJ, 348, 2272. 10.1136/bmj.g2272 PMC3987052 24736206

[fsn32299-bib-0081] Morena, M., De Castro, V., Gray, J. M., Palmery, M., Trezza, V., Roozendaal, B., Hill, M. N., & Campolongo, P. (2015). Training‐associated emotional arousal shapes endocannabinoid modulation of spatial memory retrieval in rats. Journal of Neuroscience, 35(41), 13962–13974. 10.1523/JNEUROSCI.1983-15.2015 26468197 PMC6608184

[fsn32299-bib-0082] Mori, T. A. (2014). Dietary *n*‐3 PUFA and CVD: A review of the evidence. The Proceedings of the Nutrition Society, 73(1), 57–64. 10.1017/S0029665113003583 24119287

[fsn32299-bib-0083] Mori, T. A., & Beilin, L. J. (2004). Omega‐3 fatty acids and inflammation. Current Atherosclerosis Reports, 6(6), 461–467. 10.1007/s11883-004-0087-5 15485592

[fsn32299-bib-0084] Mori, T. A., Burke, V., Puddey, I. B., Watts, G. F., O’Neal, D. N., Best, J. D., & Beilin, L. J. (2000). Purified eicosapentaenoic and docosahexaenoic acids have differential effects on serum lipids and lipoproteins, LDL particle size, glucose, and insulin in mildly hyperlipidemic men. The American Journal of Clinical Nutrition, 71(5), 1085–1094. 10.1016/S0021-9150(00)80500-6 10799369

[fsn32299-bib-0085] Mori, T. A., Watts, G. F., Burke, V., Hilme, E., Puddey, I. B., & Beilin, L. J. (2000). Differential effects of eicosapentaenoic acid and docosahexaenoic acid on vascular reactivity of the forearm microcirculation in hyperlipidemic, overweight men. Circulation, 102(11), 1264–1269. 10.1161/01.CIR.102.11.1264 10982541

[fsn32299-bib-0086] Mozaffarian, D., & Wu, J. H. Y. (2011). Omega‐3 fatty acids and cardiovascular disease: Effects on risk factors, molecular pathways, and clinical events. Journal of the American College of Cardiology, 58(20), 2047–2067. 10.1016/j.jacc.2011.06.063 22051327

[fsn32299-bib-0087] Mozaffarian, D., & Wu, J. H. Y. (2012). (*n*‐3) Fatty acids and cardiovascular health: Are effects of EPA and DHA shared or complementary? Journal of Nutrition, 142(3), 614–625. 10.3945/jn.111.149633 PMC3278271 22279134

[fsn32299-bib-0088] Muldoon, M. F., Ryan, C. M., Sheu, L., Yao, J. K., Conklin, S. M., & Manuck, S. B. (2010). Serum phospholipid docosahexaenonic acid is associated with cognitive functioning during middle adulthood. The Journal of Nutrition, 140(4), 848–853. 10.3945/jn.109.119578 20181791 PMC2838625

[fsn32299-bib-0089] National Health and Medical Research Council, Australian Government Department of Health and Ageing, New Zealand Ministry of Health . (2006). Nutrient reference values for Australia and New Zealand including recommended dietary intakes. National Health and Medical Research Council.

[fsn32299-bib-0090] Nestel, P., Shige, H., Pomeroy, S., Cehun, M., Abbey, M., & Raederstorff, D. (2002). The *n*‐3 fatty acids eicosapentaenoic acid and docosahexaenoic acid increase systemic arterial compliance in humans. American Journal of Clinical Nutrition, 76(2), 326–330. 10.1093/ajcn/76.2.326 12145002

[fsn32299-bib-0091] Olsen, S. F., Secher, N. J., Tabor, A., Weber, T., Walker, J. J., & Gluud, C. (2000). Randomised clinical trials of fish oil supplementation in high risk pregnancies. An International Journal of Obstetrics and Gynaecology, 107(3), 382–395. 10.1111/j.1471-0528.2000.tb13235.x 10740336

[fsn32299-bib-0092] Parada, J., & Aguilera, J. M. (2007). Food microstructure affects the bioavailability of several nutrients. Journal of Food Science, 72(2), 21–32. 10.1111/j.1750-3841.2007.00274.x 17995848

[fsn32299-bib-0093] Raatz, S. K., Redmon, J. B., Wimmergren, N., Donadio, J. V., & Bibus, D. M. (2009). Enhanced absorption of *n*‐3 fatty acids from emulsified compared with encapsulated fish oil. Journal of the American Dietetic Association, 109(6), 1076–1081. 10.1016/j.jada.2009.03.006 19465191 PMC2701654

[fsn32299-bib-0094] Rauch, B., Schiele, R., Schneider, S., Diller, F., Victor, N., Gohlke, H., Gottwik, M., Steinbeck, G., Del Castillo, U., Sack, R., Worth, H., Katus, H., Spitzer, W., Sabin, G., Senges, J., & OMEGA Study Group . (2010). OMEGA, a randomized, placebo‐controlled trial to test the effect of highly purified omega‐3 fatty acids on top of modern guideline‐adjusted therapy after myocardial infarction. Circulation, 122(21), 2152–2159. 10.1161/CIRCULATIONAHA.110.948562 21060071

[fsn32299-bib-0095] Rogers, P. J., Appleton, K. M., Kessler, D., Peters, T. J., Gunnell, D., Hayward, R. C., Heatherley, S. V., Christian, L. M., Mcnaughton, S. A., & Ness, A. R. (2008). No effect of *n*‐3 long‐chain polyunsaturated fatty acid (EPA and DHA) supplementation on depressed mood and cognitive function: A randomised controlled trial. British Journal of Nutrition, 99(2), 421–431. 10.1017/S0007114507801097 17956647

[fsn32299-bib-0096] Roman, A. S., Schreher, J., Mackenzie, A. P., & Nathanielsz, P. W. (2006). Omega‐3 fatty acids and decidual cell prostaglandin production in response to the inflammatory cytokine IL‐1β. American Journal of Obstetrics and Gynecology, 195(6), 1693–1699. 10.1016/j.ajog.2006.04.009 16792994

[fsn32299-bib-0097] Rossmeisl, M., Jilkova, Z. M., Kuda, O., Jelenik, T., Medrikova, D., Stankova, B., Kristinsson, B., Haraldsson, G. G., Svensen, H., Stoknes, I., Sjövall, P., Magnusson, Y., Balvers, M. G., Verhoeckx, K. C., Tvrzicka, E., Bryhn, M., & Kopecky, J. (2012). Metabolic effects of *n*‐3 PUFA as phospholipids are superior to triglycerides in mice fed a high‐fat diet: Possible role of endocannabinoids. PLoS One, 7(6), 1–13. 10.1371/journal.pone.0038834 PMC3372498 22701720

[fsn32299-bib-0098] Salem, N., & Eggersdorfer, M. (2015). Is the world supply of omega‐3 fatty acids adequate for optimal human nutrition? Current Opinion in Clinical Nutrition and Metabolic Care, 18(2), 147–154. 10.1097/MCO.0000000000000145 25635599

[fsn32299-bib-0099] Salem, N., & Kuratko, C. N. (2014). A reexamination of krill oil bioavailability studies. Lipids in Health and Disease, 13, 137. 10.1186/1476-511X-13-137 25156381 PMC4161905

[fsn32299-bib-0100] Schmidt, E. B., Arnesen, H., De Caterina, R., Rasmussen, L. H., & Kristensen, S. D. (2005). Marine *n*‐3 polyunsaturated fatty acids and coronary heart disease: Part I. Background, epidemiology, animal data, effects on risk factors and safety. Thrombosis Research, 115(3), 163–170. 10.1016/j.thromres.2004.09.006 15617737

[fsn32299-bib-0101] Schmidt, E. B., Kristensen, S. D., De Caterina, R., & Roger Illingworth, D. (1993). The effects of *n*‐3 fatty acids on plasma lipids and lipoproteins and other cardiovascular risk factors in patients with hyperlipidemia. Atherosclerosis, 103(2), 107–121. 10.1016/0021-9150(93)90254-R 8292089

[fsn32299-bib-0102] Schuchardt, J. P., & Hahn, A. (2013). Bioavailability of long‐chain omega‐3 fatty acids. Prostaglandins Leukotrienes and Essential Fatty Acids, 89(1), 1–8. 10.1016/j.plefa.2013.03.010 23676322

[fsn32299-bib-0103] Seddon, J. M., George, S., & Rosner, B. (2006). Cigarette smoking, fish consumption, omega‐3 fatty acid intake, and associations with age‐related macular degeneration: The US twin study of age‐related macular degeneration. Archives of Ophthalmology, 124(7), 995–1001. 10.1001/archopht.124.7.995 16832023

[fsn32299-bib-0104] Serhan, C. N., Chiang, N., & Van Dyke, T. E. (2008). Resolving inflammation: Dual anti‐inflammatory and pro‐resolution lipid mediators. Nature Reviews. Immunology, 8(5), 349–361. 10.1038/nri2294 PMC2744593 18437155

[fsn32299-bib-0105] Shahidi, F., & Ambigaipalan, P. (2018). Omega‐3 polyunsaturated fatty acids and their health benefits. Annual Review of Food Science and Technology, 9, 345–381. 10.1146/annurev-food-111317-095850 29350557

[fsn32299-bib-0106] Sherry, C. L., Oliver, J. S., & Marriage, B. J. (2015). Docosahexaenoic acid supplementation in lactating women increases breast milk and plasma docosahexaenoic acid concentrations and alters infant omega 6:3 fatty acid ratio. Prostaglandins Leukotrienes and Essential Fatty Acids, 95, 63–69. 10.1016/j.plefa.2015.01.005 25701002

[fsn32299-bib-0107] Smit, E. N., Oelen, E. A., Seerat, E., Muskiet, F. A., & Boersma, E. R. (2000). Breast milk docosahexaenoic acid (DHA) correlates with DHA status of malnourished infants. Archives of Disease in Childhood, 82(6), 493–494. 10.1136/adc.82.6.493 10833187 PMC1718344

[fsn32299-bib-0108] Sposito, A. C., Caramelli, B., Fonseca, F. A. H., Bertolami, M. C., Rassi, A. Jr, Neto, A. A., Souza, A. D., Lottenberg, A. M. P., Chacra, A. P., Faludi, A. A., Loures‐Vale, A. A., Carvalho, A. C., Duncan, B., Gelonese, B., Polanczyk, C., Sobrinho, C. R. M. R., Scherr, C., Karla, C., Armaganijan, D., … Salgado, W. (2007). IV Brazilian guideline for dyslipidemia and atherosclerosis prevention: Department of atherosclerosis of Brazilian society of cardiology. Arquivos Brasileiros De Cardiologia, 88(1), 2–19. 10.1590/s0066-782x2007000700002 17515982

[fsn32299-bib-0109] Stonehouse, W., Conlon, C. A., Podd, J., Hill, S. R., Minihane, A. M., Haskell, C., & Kennedy, D. (2013). DHA supplementation improved both memory and reaction time in healthy young adults: A randomized controlled trial. American Journal of Clinical Nutrition, 97(5), 1134–1143. 10.3945/ajcn.112.053371 23515006

[fsn32299-bib-0110] Swanson, D., Block, R., & Mousa, S. A. (2012). Omega‐3 fatty acids EPA and DHA: Health benefits throughout life. Advances in Nutrition, 3(1), 1–7. 10.3945/an.111.000893 22332096 PMC3262608

[fsn32299-bib-0111] Tambakhe, M. K., & Pawar, P. A. (2014). Supplementation of infant formula with Probiotics, Prebiotics, DHA & ARA: A systematic review. Journal of Food and Nutrition Sciences, 2(4), 185. 10.11648/j.jfns.20140204.24

[fsn32299-bib-0112] The French Food Safety Agency . (2010). Opinion of the French Food Safety Agency on the update of French population reference intakes for fatty acids. https://www.anses.fr/en/system/files/NUT2006sa0359EN.pdf

[fsn32299-bib-0113] The Writing Committee of the Report on Cardiovascular Health and Diseases in China . (2020). Report on cardiovascular health and diseases in China 2019: An updated summary. Chinese Circulation Journal, 35(9), 833–854. 10.3969/j.issn.1000-3614.2020.09.001

[fsn32299-bib-0114] Thien, F. C., De Luca, S., Woods, R. K., & Abramson, M. J. (2000). Dietary marine fatty acids (fish oil) for asthma in adults and children. Cochrane Database of Systematic Reviews, 2, CD001283. 10.1002/14651858.cd001283 12137622

[fsn32299-bib-0115] Titova, O. E., Sjögren, P., Brooks, S. J., Kullberg, J., Ax, E., Kilander, L., Riserus, U., Cederholm, T., Larsson, E. M., Johansson, L., Ahlström, H., Lind, L., Schiöth, H. B., & Benedict, C. (2013). Dietary intake of eicosapentaenoic and docosahexaenoic acids is linked to gray matter volume and cognitive function in elderly. Age, 35(4), 1495–1505. 10.1007/s11357-012-9453-3 22791395 PMC3705118

[fsn32299-bib-0116] Tou, J. C., Altman, S. N., Gigliotti, J. C., Benedito, V., & Cordonier, E. L. (2011). Different sources of omega‐3 polyunsaturated fatty acids affects apparent digestibility, tissue deposition, and tissue oxidative stability in growing female rats. Lipids in Health and Disease, 10(1), 179. 10.1186/1476-511X-10-179 21999902 PMC3216256

[fsn32299-bib-0117] Tou, J. C., Jaczynski, J., & Chen, Y. C. (2007). Krill for human consumption: Nutritional value and potential health benefits. Nutrition Reviews, 65(2), 63–77. 10.1111/j.1753-4887.2007.tb00283.x 17345959

[fsn32299-bib-0118] Uauy, R., Mena, P., & Rojas, C. (2000). Essential fatty acids in early life: Structural and functional role. The Proceedings of the Nutrition Society, 59(1), 3–15. 10.1017/S0029665100000021 10828169

[fsn32299-bib-0119] Udell, T., & Eden, B. (2008). Summary of evidence: Fish, fish oils, n‐3 polyunsaturated fatty acids and cardiovascular health. Heart Foundation of Australia.

[fsn32299-bib-0120] Ulven, S. M., Kirkhus, B., Lamglait, A., Basu, S., Elind, E., Haider, T., Berge, K., Vik, H., & Pedersen, J. I. (2011). Metabolic effects of krill oil are essentially similar to those of fish oil but at lower dose of EPA and DHA, in healthy volunteers. Lipids, 46(1), 37–46. 10.1007/s11745-010-3490-4 21042875 PMC3024511

[fsn32299-bib-0121] Valenzuela, A., Valenzuela, V., Sanhueza, J., & Nieto, S. (2005). Effect of supplementation with docosahexaenoic acid ethyl ester and sn‐2 docosahexaenyl monoacylglyceride on plasma and erythrocyte fatty acids in rats. Annals of Nutrition and Metabolism, 49(1), 49–53. 10.1159/000084177 15735367

[fsn32299-bib-0122] Van Der Westhuyzen, J., Chetty, N., & Atkinson, P. M. (1988). Fatty acid composition of human milk from South African black mothers consuming a traditional maize diet. European Journal of Clinical Nutrition, 42(3), 213–220. 3383825

[fsn32299-bib-0123] Vandal, M., Freemantle, E., Tremblay‐Mercier, J., Plourde, M., Fortier, M., Bruneau, J., Gagnon, J., Begin, M., & Cunnane, S. C. (2008). Plasma omega‐3 fatty acid response to a fish oil supplement in the healthy elderly. Lipids, 43(11), 1085–1089. 10.1007/s11745-008-3232-z 18795357

[fsn32299-bib-0124] Watson, H., Mitra, S., Croden, F. C., Taylor, M., Wood, H. M., Perry, S. L., Spencer, J. A., Quirke, P., Toogood, G. J., Lawton, C. L., Dye, L., Loadman, P. M., & Hull, M. A. (2018). A randomised trial of the effect of omega‐3 polyunsaturated fatty acid supplements on the human intestinal microbiota. Gut, 67(11), 1974–1983. 10.1136/gutjnl-2017-314968 28951525

[fsn32299-bib-0125] Willatts, P., Forsyth, S., Agostoni, C., Casaer, P., Riva, E., & Boehm, G. (2016). Effects of long‐chain PUFA supplementation in infant formula on cognitive function in later childhood. World Review of Nutrition and Dietetics, 114, 71–72. 10.1159/000441812 23783296

[fsn32299-bib-0126] Yang, L. Y., Kuksis, A., & Myher, J. J. (1990). Intestinal absorption of menhaden and rapeseed oils and their fatty acid methyl and ethyl esters in the rat. Biochemistry and Cell Biology, 68(2), 480–491. 10.1139/o90-068 2344400

[fsn32299-bib-0127] Younge, N., Yang, Q., & Seed, P. C. (2017). Enteral high fat‐polyunsaturated fatty acid blend alters the pathogen composition of the intestinal microbiome in premature infants with an enterostomy. The Journal of Pediatrics, 181(2), 93–101. 10.1016/j.jpeds.2016.10.053 27856001 PMC5274578

[fsn32299-bib-0128] Yuhas, R., Pramuk, K., & Lien, E. L. (2006). Human milk fatty acid composition from nine countries varies most in DHA. Lipids, 41(9), 851–858. 10.1007/s11745-006-5040-7 17152922

[fsn32299-bib-0129] Yurko‐Mauro, K., Alexander, D. D., & Van Elswyk, M. E. (2015). Docosahexaenoic acid and adult memory: A systematic review and meta‐analysis. PLoS One, 10(3), 1–18. 10.1371/journal.pone.0120391 PMC4364972 25786262

[fsn32299-bib-0130] Zhang, P., Lavoie, P. M., Lacaze‐Masmonteil, T., Rhainds, M., & Marc, I. (2014). Omega‐3 long‐chain polyunsaturated fatty acids for extremely preterm infants: A systematic review. Pediatrics, 134(1), 120–134. 10.1542/peds.2014-0459 24913791

